# Delirium Superimposed on Dementia in Perioperative Period and Intensive Care

**DOI:** 10.3390/jcm9103279

**Published:** 2020-10-13

**Authors:** Łukasz J. Krzych, Natalia Rachfalska, Zbigniew Putowski

**Affiliations:** 1 Department of Anaesthesiology and Intensive Care, Faculty of Medical Sciences in Katowice, Medical University of Silesia, 14 Medyków Street, 40752 Katowice, Poland; 2 Students’ Scientific Society, Department of Anaesthesiology and Intensive Care, Faculty of Medical Sciences in Katowice, Medical University of Silesia, 40752 Katowice, Poland; n.rachfalska@gmail.com (N.R.); putowski.zbigniew@gmail.com (Z.P.)

**Keywords:** dementia, delirium, delirium superimposed on dementia, perioperative care, critical care

## Abstract

Delirium is a life-threatening condition, the causes of which are still not fully understood. It may develop in patients with pre-existing dementia. Delirium superimposed on dementia (DSD) can go completely unnoticed with routine examination. It may happen in the perioperative period and in the critical care setting, especially in the ageing population. Difficulties in diagnosing and lack of specific pharmacological and non-pharmacological treatment make DSD a seriously growing problem. Patient-oriented, multidirectional preventive measures should be applied to reduce the risk of DSD. For this reason, anesthesiologists and intensive care specialists should be aware of this interesting condition in their everyday clinical practice.

## 1. Introduction

Delirium is a serious clinical condition characterized by a sudden attention disruption and cognitive decline, the course of which fluctuates, and which cannot be explained by the earlier occurrence of neurocognitive disorders. These disturbances must not occur in states of disturbed consciousness, such as coma. Delirium is considered as a diffuse brain injury type II with cognitive abilities, causing a decompensation of functions of the central nervous system (CNS). By contrast, the major neurocognitive disorders, often described with a single term “dementia,” are significant cognitive impairments relative to baseline state, in at least one area that affects the patient’s independence and cannot be better explained by the occurrence of another mental disorder. Major neurocognitive disorders must occur independently, except for a possible delirium incident. To simplify the terminology, the researchers often use the term “dementia” to describe this vague state [1].

The above-mentioned facts clearly show that the convergence of definitions can lead to problems in differentiating between delirium and dementia, especially in patients admitted to the emergency department (ER) or intensive care unit (ICU), and in other situations when contact with a caregiver or family member is difficult or even impossible to establish. The problem seems all the more significant because Elie et al. estimated that in patients with oncological diseases, more than 57% of cases of delirium were not detected in the initial assessment [2]. In addition, delirium and dementia can occur simultaneously. Both of these disorders increase each other’s risk of development [3,4] and are more common among the elderly [1,5]. Both delirium and dementia have a common pathological mechanism, which results in many similarities in the clinical presentation, creating additional diagnostic difficulties [6].

Demographic projections in developed countries suggest that the number of people over 60 years of age will grow substantially in the near future [7] and therefore, the problems associated with diagnosing patients with delirium superimposed on dementia (DSD) will also increase. Delirium occurs in 8–17% of patients admitted to EDs and 7–50% of patients admitted to the ICUs. The incidence of new cases of delirium during hospitalization ranges from 11% in internal medicine wards to 82% in ICUs [8]. In 2019, according to Alzheimer Europe Initiative, the estimated prevalence of dementia in Poland was 525,084 patients (i.e., 1.38% of the population), while by 2050 it is expected to reach more than 1 million patients (i.e., 3.23% of the population) [9]. The steady ageing of the population makes neurocognitive disorders a common occurrence in all hospital wards.

Delirium occurs in 22–89% of patients with dementia in hospitals and nursing homes [10]. Recognizing delirium superimposed on dementia is crucial because DSD prolongs the hospitalization period and increases mortality compared to sole delirium [11]. Each incident of delirium increases the costs of hospitalization; in the United States, it leads to an estimated increase from 16 to 64 thousand dollars per patient [12]. This is all the more crucial, considering reports that delirium can be avoided in about 30–40% of cases [13,14].

DSD diagnostics and treatment may be challenging even for experienced clinicians, especially non-psychiatrists. Therefore, anesthesiologists and intensive care specialists should familiarize themselves with this serious clinical problem, which may develop in the elderly in the perioperative period and during critical illness. The aim of this paper is to present basic data on pathophysiology, diagnostics, and prevention/treatment of dementia and delirium, which may be helpful to manage DSD in the perioperative period.

## 2. Pathophysiology of Dementia

Dementia is an umbrella term for a collection of symptoms that are caused by a direct brain disease and are characterized by clinically numerous disorders of higher cortical functions, including memory, thinking, orientation, understanding, numeracy, learning capacity, language skills, and judgment. Dementia can be classified as either primary, which is most often caused by other neurodegenerative diseases, or secondary, which is caused by known causes, such as cerebral circulatory disorders (e.g., vascular dementia—VaD), or infections [15]. The common denominator of all dementia diseases is the disappearance of neurons and the loss of connections between them [16].

**Figure 1:**
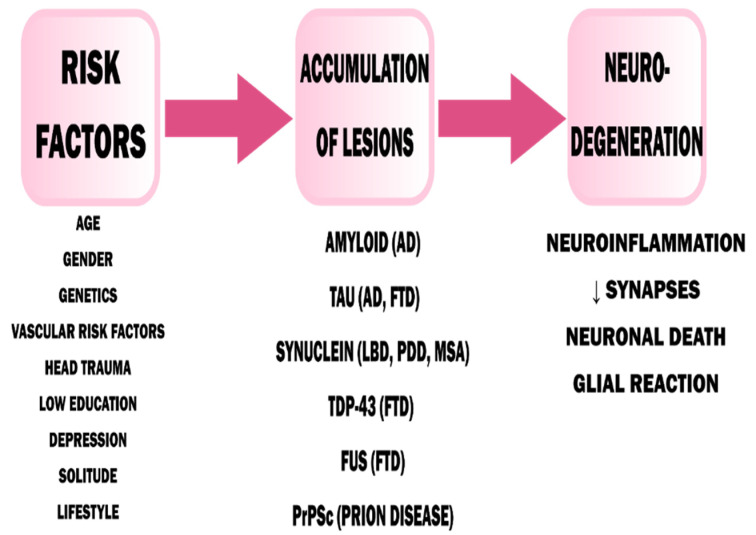
Pathophysiology of dementia [17]. AD—Alzheimer’s Disease, FTD—Frontotemporal Dementia, LBD—Lewy Body Dementia, PDD—Parkinson’s Disease Dementia, MSA—Multiple System Atrophy. Adapted from Saladrini, A., Seminars in Neurology; published by Thieme Medical Publishers, 2019.

In primary dementia, the cause of nerve cell degeneration is most often (but not in all cases) the deposition of proteins in the brain, having a pathological β-sheet structure—in the case of Alzheimer’s disease (AD), it is amyloid-β, for dementia with Lewy bodies (DLB) and dementia in Parkinson’s disease (PDD) α-synuclein [16,17]. These deposits provoke an inflammatory reaction and activation of microglia through TLRs (Toll-like receptors) [18]. This drives the course of neuroinflammation and contributes to the chronic course of the disease (Figure 1) [19]. However, the initial loss of neurons is clinically asymptomatic because of the large cognitive reserve of the CNS [16].

In secondary dementia syndromes, neurons die as a result of another medical condition. Most often, secondary dementia has vascular etiology, for example, as a result of advanced atherosclerosis of vessels supplying the CNS. Another secondary cause of dementia is infection, especially viral one [20].

## 3. Pathophysiology of Delirium

**Figure 2:**
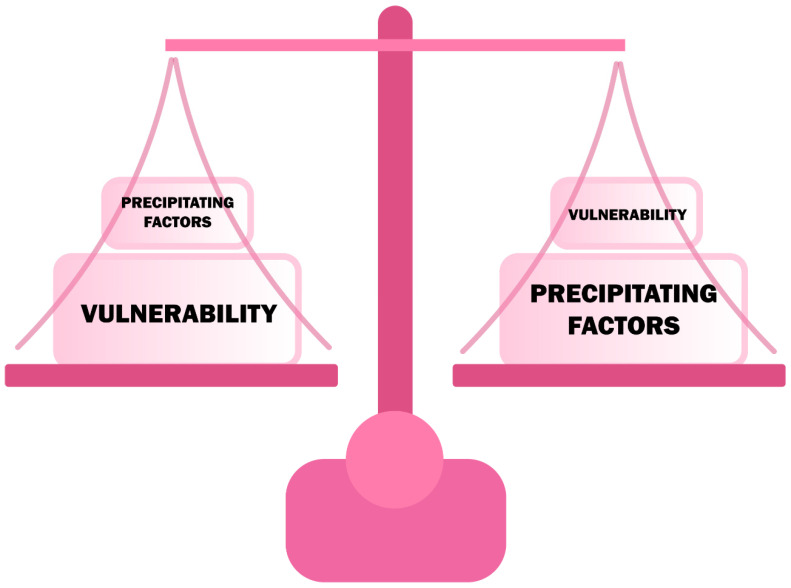
The relationship of vulnerability and precipitating factors in developing delirium.

Delirium is one of the most common, urgent neuropsychiatric syndromes that usually develops acutely or subacutely in response to another sudden pathological condition in the body [21]. The increased occurrence of delirium among elderly people may be explained by generalized frailty, a decrease in acetylcholinergic neurons, a decrease in the level of aerobic metabolism in the CNS, and an age-related increase in stress-related neurotransmitters [22]. The causes of delirium are divided into the so-called precipitant factors and the vulnerability factors [23], which are listed in Table 1. Their interdependence means that a patient with high vulnerability can develop delirium with a slight increase in precipitant factors [23] (Figure 2). Infections (16–67% of cases) are the most commonly mentioned precipitant factors, while dementia is most consistently mentioned among the vulnerability factors [8,24,25,26]. Precipitating factors significantly exaggerate the risk of delirium in the perioperative period, in patients at risk or with developed dementia.

Theories about the pathomechanism of delirium vary and it is unlikely that only one mechanism is responsible for such a broad spectrum of symptoms. A common hypothesis explaining the symptoms and the predisposition of delirium in people with Alzheimer’s disease is the theory of cholinergic deficiency. Evidence of its validity is the observed incidence of delirium in people taking substances with anticholinergic action [30,31]. The synthesis of acetylcholine is closely related to the aerobic metabolism of carbohydrates, and therefore in the state of hypoxia and hypoglycemia, cholinergic activity decreases [32]. This explains why delirium occurs in a wide spectrum of medical emergencies.

Other neurotransmitter disturbances that occur with delirium concern dopamine and γ-aminobutyric acid (GABA). It has been noted that dopaminergic substances can provoke a hyperactive form of delirium [33,34,35,36], indicating the possible role of excess dopamine release. Importantly, benzodiazepine drugs that increase GABA-ergic stimulation are associated with a higher risk of delirium [37,38].

The hypothesis associated with the aging of neural tissue explains well the higher incidence of delirium in people over 65 years of age. This hypothesis assumes that with neural aging, the proportion of neurotransmitters involved in stress regulation changes the vascularization and, in addition, diminishes the overall number of neurons [29,39]. Moreover, with age, the level of pro-inflammatory mediators increases [40] and so does the accumulation of reactive oxygen species [41]. In conclusion, with age, stress tolerance and neural reserve decreases which, in situations of medical emergencies, contribute to the collapse of cognitive stability.

**Figure 3:**
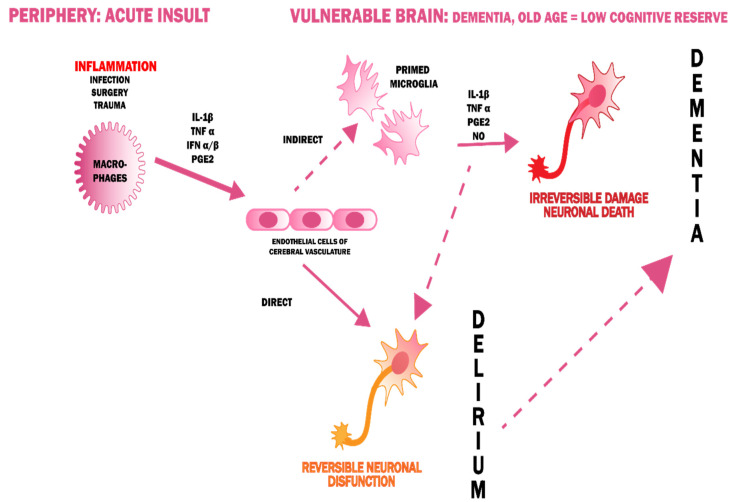
A possible pathogenesis of delirium, linking it with dementia [50].

The theory of inflammation assumes that delirium is a manifestation of a systemic disease that crosses the blood-brain barrier (BBB) [34,42,43,44]. This is evidenced by the presence of elevated markers of inflammation in patients with either delirium [45,46] or dementia [47] which indicates the interlocking pathomechanisms of both of these conditions. Pro-inflammatory cytokines, entering the CNS because of damage to the BBB, cause microglia activation. The inflammation is accompanied by oxidative stress because of the high content of lipids (myelin sheaths), high levels of aerobic metabolism, and a low reserve of antioxidant factors [48]. This results in a disruption of synaptic transmission and the occurrence of characteristic neuropsychiatric symptoms in patients with delirium (Figure 3) [49]. Chronic inflammatory activity of microglia in neurodegenerative diseases can be a predisposing factor to the increased response in the event of systemic inflammation, that is, to the onset of delirium. Therefore, this theory forges a strong link between delirium and dementia.

## 4. Delirium and Dementia—A Pathophysiological Interrelationship

**Figure 4:**
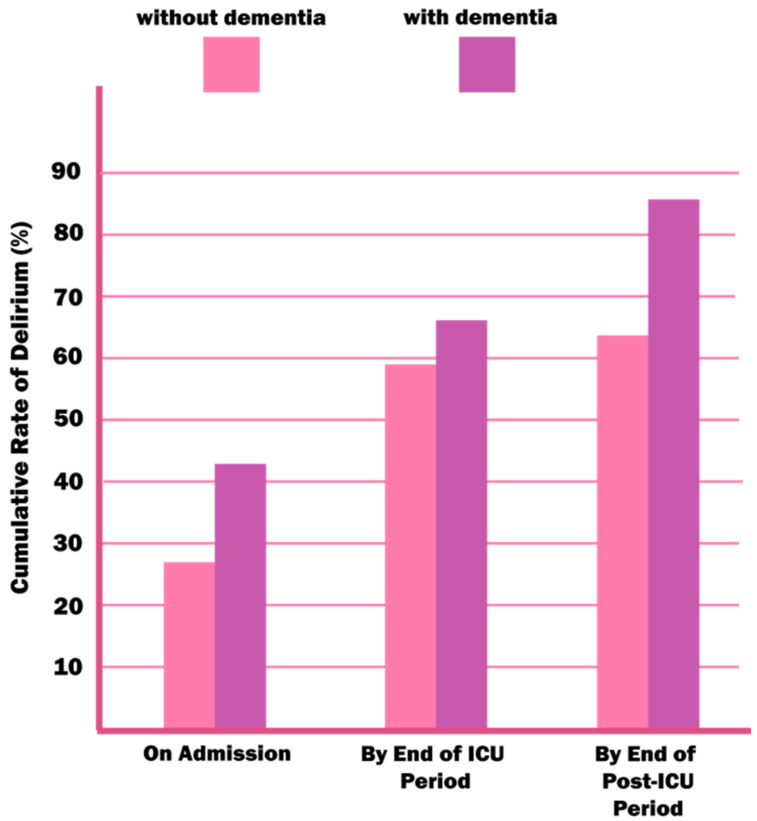
An estimated rate of delirium in patients with and without dementia in the ICU environment. Reproduced with permission from McNicoll L, Pisani MA, Zhang Y, Ely EW, Siegel MD, Inouye SK, Journal of the American Geriatrics Society, published by Wiley Online Library, 2003.

The mutual correlation between delirium and dementia is indisputable—the neuroinflammation, the oxidative stress, neurotransmitters, and brain aging theories bind the two together. The way in which delirium and dementia affect each other has been the subject of many debates. Delirium could be an early marker of susceptibility to future dementia or an early manifestation of preclinical loss of cognitive function. In addition, the occurrence of delirium alone could lead to a permanent damage of neurons, which would contribute to the development of dementia [3,51]. On the other hand, dementia is a proven risk factor for delirium (Figure 4) because of reduced cognitive reserve [8,25,26,28,29,52].

## 5. Symptoms of Delirium

Symptoms of delirium in people with neurocognitive disorders do not differ much from the delirium found in people without baseline cognitive disorders. Moreover, dementia can overlap with pre-existing symptoms of dementia which is often a diagnostic obstacle.

Delirium is dominated by distortions of attention and awareness (criterion A), and accompanied by changes in basic cognitive performance (criterion C). Delirium generally develops rapidly—within a several hours or a few days (criterion B).

A distortion of attention (criterion A) is understood as a reduced ability to target, focus, maintain, and shift attention. This symptom is quite characteristic because in dementia, attention usually remains undisturbed [53].

Disturbance of awareness (criterion A) is manifested by an incorrect orientation in the environment, time, and even self. It develops rapidly, usually within a few hours, it is usually expressed to the fullest in the evening and at night.

Attention and awareness disorders are accompanied by disruptions in at least one cognitive domain among the following (criterion C): learning capacity, memory, orientation, language skills, perception, visual-motor functions. It should be noted that cognitive function testing can only be carried out at an appropriate level of consciousness, which allows for the response to verbal stimuli, i.e., the exclusion criteria include patients in comas (criterion D) [1].

Arousal, defined as the level of sensory stimuli necessary to maintain the patient’s attention at the time of issuing commands/asking questions, is an important criterion in the differential diagnosis of dementia and delirium [54]. Attention and cognitive function can be impaired in dementia, especially advanced stage, but arousal usually remains unchanged [55]. The explanation for this is the fact that in order to maintain proper attention, the reticular system of the brain stem must remain intact. With dementia, the part of the brain that is mostly dysfunctional is the cortex [56]. Delirium, on the other hand, is caused by a systemic condition that affects the CNS as a whole.

Other common disruptions in delirium, which are less likely to be confused with “pure” dementia, include circadian rhythm disorders, delusions, and emotional changes. Most clinical features that distinguish those diseases from each other are listed in Table 2. Delirium superimposed on dementia can be manifested by the occurrence of the phenomenon called sundowning syndrome, which is expressed as behavioral and cognitive deterioration in the evening and at night [57].

## 6. Types of Delirium

Despite the main characteristics of delirium, it should be emphasized that it is, in and of itself, heterogeneous. There are four phenotypes of delirium that have been distinguished. Subclinical delirium which, although it does not present clear symptoms, prolongs the period of hospitalization, increases mortality after leaving the hospital, and reduces the functional and cognitive performance of patients [60]. Hypoactive delirium, which is often undiagnosed [61] and has the worst prognosis, is the most common among older patients [62,63,64,65]. Patients with hypoactive delirium are described as sleepy and lethargic. Hyperactive delirium, which is easier to diagnose, causes patients to have an increased psychomotor activity, they seem restless, agitated, concerned, and often aggressive (Table 3). However, it happens less often among older patients than the hypoactive type. Mixed delirium is characterized by the alternating occurrence of hypo- and hyperactive type. In the perioperative period, hyperactive delirium is the most frequently described in the literature, however, the hypoactive delirium is believed to be significantly underdiagnosed.

## 7. Diagnosis of Delirium Superimposed on Dementia

Because of the fact that, among the elderly, the predominant type of delirium is the hypoactive variant, the problems associated with diagnosing it are colossal, leading to a large number of cases which go undiagnosed. The reasons why hypoactive delirium often goes undiagnosed are as follows [66]:

1. Loneliness of the patient and absence of a person that would monitor the patient’s daily condition and who could notice a sudden change in behavior;
2. Lack of doctor–patient communication;
3. Misconception that older people are “withdrawn” and disorganized on their own;
4. Misconception that a patient being asleep is only attributable to fatigue.

While establishing a diagnosis of delirium, all criteria contained in Diagnostic and Statistical Manual of Mental Disorders 5th Edition (DSM-V) should be evaluated. The most popular screening test used worldwide is CAM (*Confusion Assessment Method*), a tool created by Dr. Sharon Inouye that can be used by medical staff without a psychiatric education [67]. The CAM algorithm is based on the presence of the main features of delirium: (1) sudden onset or fluctuation of symptoms, (2) attention disturbances, (3) disorganized thought process, and (4) impaired consciousness. Moreover, it investigates the presence of other cognitive disorders such as memory loss, impaired perception (e.g., delusion), agitation, psychomotor slowing, and disturbed circadian rhythm. The CAM test takes less than 5 min to complete, and its sensitivity and specificity to detect delirium are very high (91–97% and 85–94%, respectively) [67]. It can be successfully applied in conscious patients perioperatively.

For the needs of patients in the ICU, especially those intubated and mechanically ventilated, an adaptation of the CAM algorithm was created, which is used for diagnosing delirium in critically ill patients—CAM-ICU. It assesses four main features of delirium: (1) sudden onset or variable course, (2) inability to focus attention, (3) a changed state of consciousness—on the RASS scale, (4) a disorganized course of thinking.

Sudden onset or fluctuation of delirium (1) can be assessed by observing the patient and collecting a detailed medical history. Information about the patient’s initial mental state can be obtained from a collateral historian (e.g., family). However, such a person is only available in about 50% of cases [68]. In subsequent CAM assessments, we refer to the same initial mental state, unless, during hospitalization, there has been permanent change in the state of consciousness (e.g., stroke, hypoxia) [69]. In order for fluctuations to meet the CAM criteria, the patient must “switch” between the states of consciousness at least two times (full cycle) [69]. It should be remembered that a change in the patient’s mental state may be due to the use of sedative drugs, but the change should never be neglected in the CAM evaluation.

Attention disorders (2) should be deeply examined. There is no optimal method to assess attention disturbances when it comes to delirium. The most commonly used neuropsychological tests (in the CAM test) are: digit span, shape span, months of the year backwards (MOTYB), and serial 7 [70]. Among these tests, MOTYB shows the greatest sensitivity and ability to distinguish patients with delirium from those with dementia [71]. These tests are usually impossible to perform in ICU patients, and thus “squeezing clinicians hand on the letter A” is used in CAM-ICU algorithm.

A disturbed state of consciousness or level of arousal (3) is determined by the observation of the patient. Any non-alert state indicates a disturbed state of consciousness [72]. This can be assessed using the RASS (*Richmond Agitation-Sedation Scale*) scale. A score other than 0 (0 stands for an alert and a calm patient) indicates a disorder. Results below 0 indicate lower awareness, while above 0 indicate an agitated state [28].

**Figure 5:**
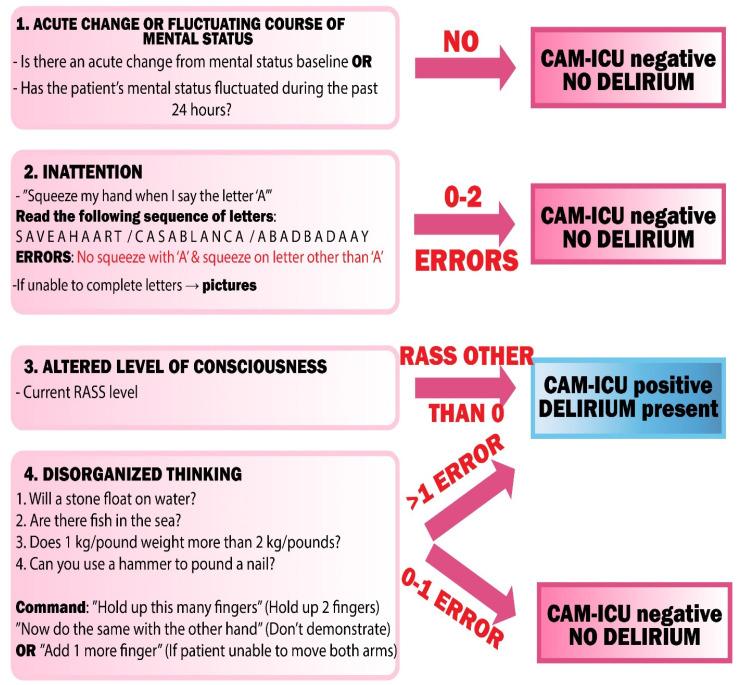
CAM-ICU flowsheet. CAM-ICU—Confusion Assessment Method—Intensive Care Unit, RASS—Richmond Agitation Sedation Scale.

A disorganized thought process (4) is indicated by chaotic and incomprehensible answers to questions asked. It can also manifest itself in a patient’s rapid change of a subject during a conversation. To assess the disorganized thought process, the patient must be able to speak or write, and therefore cannot be applied in intubated patients. Examples of questions that can be asked to a patient are summarized in Figure 5.

Additional cognitive impairment evaluated by CAM is described as the existence of the following disorders: disorientation, memory impairment, perceptual disturbances, altered sleep-wake cycle, and psychomotor retardation or agitation. It is not evaluated using the CAM-ICU.

To recognize delirium via the CAM algorithm, the patient must meet the criterion of sudden onset or fluctuation in their mental status (1), attention disturbances (2) and at least one of the following: disorganized thought flow (3) and/or altered state of consciousness (4).

Problems in diagnosing delirium superimposed on dementia by standard methods are due to the overlapping symptoms of these disease units. Attention disturbances are a hallmark symptom of delirium, but the standard tests also asses other cognitive functions, which may already be impaired in dementia syndromes. Therefore, people with dementia, despite a lack of delirium, can receive positive tests results used in the diagnosis of delirium [73]. In addition, in advanced stages of dementia, tests to assess changes in cognition do not provide sufficient information to establish diagnosis when delirium is suspected [56]. A study comparing patients with “pure delirium,” “pure dementia,” DSD, and patients without any disorders at all, proves that, when delirium occurs in a person with dementia, a deterioration in motor function can be observed [74]. For this reason, the RASS scale may be useful in diagnosing delirium in people with advanced dementia, not only in ICUs [56]. Major problems in diagnosing DSD occur in patients with Lewy bodies dementia (DLB). In such cases, cognition can fluctuate more often and perception disturbances, such as hallucination and illusions, are more common [75,76,77]. This may be crucial since introducing antipsychotic drugs in DLB patients experiencing delirium may increase drastically Parkinson’s syndrome in them. DSD may resemble other sudden conditions associated with acute deterioration of cognitive function and consciousness. In a differential diagnosis of delirium, a number of tests should be carried out because of the fact that the treatment differs depending on the conditions present. The conditions that should be considered with respect to a differential diagnosis of delirium in people with dementia are collected in Table 4 together with methods of their evaluation.

## 8. Dementia Management

Currently, there is no disease-modifying therapy proven to be successful in the management of Alzheimer’s Disease and other dementia syndromes [79,80]. The only treatment available at the moment is symptom modification and harm minimization therapy. Therapeutic interventions in dementias are classified as pharmacological or non-pharmacological [81]. As dementia is mostly reported in the elderly, it usually coexists with other diseases like ischemic heart disease, hypertension, chronic pulmonary disease, or peptic ulcer disease. A clinician should always be aware of possible interactions of frequently used medications in the elderly, as those might pose a threat greater than precognitive treatment. Below, we have focused only on basic medications used in dementia management, as those interventions may lead to possible complications in the process of treatment of patients in anesthesiology and intensive care setting.

### 8.1. Acetylcholinesterase Inhibitors

The mainstay of symptomatic treatment of Alzheimer’s Disease are acetylcholinesterase inhibitors (AChEIs). These agents increase acetylcholine availability by inhibiting its breakdown in the synapses and are effective in mild, moderate, and severe AD [82,83,84]. There is no evidence that those medications alter the course of the underlying dementing process [85]. Currently, the most commonly used drugs are: donepezil, rivastigmine, and galantamine.

It is vital to note that donepezil, out of all of the AChEIs, showed the strongest association with cases of QTc prolongation [86], and thus its use should be reconsidered in patients treated with citalopram for their depression (that often co-exist with AD) [87]. If other agents that prolong QTc are used, continuous electrocardiographic monitoring is required in the perioperative period. Rivastigmine is eliminated by renal route [83]. Clearance of rivastigmine is significantly lower in patients with severe renal impairment and in patients with liver failure. Because of that patients with moderate and severe kidney or liver diseases may be able to only tolerate lower doses of rivastigmine [88]. Galantamine is contraindicated in patients with severe hepatic impairment (Child Pugh >9 points) and/or renal impairment with creatinine clearance <9 mL/min [89]. It should be applied cautiously in subjects with or at risk of AKI.

Because of the mechanism of AChEIs that include increasing acetylcholine levels, they share the most common adverse events. Gastrointestinal adverse events, such as nausea, vomiting, dyspepsia, and diarrhea, originate from intestinal muscarinic receptor activation. They may lead to loss of bodyweight and nutritional deficiency, often causing hypoalbuminemia and anemia. Cholinesterase inhibitors may increase gastric acid secretion. Therefore, patients with risk of gastrointestinal bleeding (e.g., receiving NSAIDs or having history of peptic ulcer disease) should be closely monitored [83]. Although not as common as gastrointestinal side effects, cardiovascular adverse reactions pose danger to frail elderly patients. All of AChEIs may have vagotonic effects on the sinoatrial and atrioventricular nodes, which may manifest as bradycardia or heart block in patients with underlying cardiac abnormalities [82,83,84]. Moreover, they may cause hypotension [90] and thus lead to orthostatic falls in the elderly. Syncope has been reported in association with the use of all of those medications. Because of their cholinomimetic actions, AChEI should be thoroughly thought out in patients with a history of asthma or obstructive pulmonary disease. Cholinesterase inhibitors may have some potential to cause generalized convulsion [82,83,84].

Pharmacodynamic drug interactions pose greater clinical significance than pharmacokinetic interactions, especially in patients with dementia [91]. The most obvious interaction is interference with the activity of anticholinergic medications. An important interaction from anesthesiologist’s point of view is strong likelihood of exaggerating succinylcholine-type muscle relaxation during anesthesia. Although AChEIs are not directly contraindicated with anesthetics, discontinuation prior to surgery is advisable [92]. Based on half-lives of these agents, donepezil should be discontinued approximately 2 weeks prior to surgery, galantamine approximately 1–2 days and rivastigmine 3–4 days [92]. Considering DSD, antipsychotic use in patients with dementia is reported frequently. Some typical antipsychotics such as thioridazine are known to antagonize cholinesterase inhibitors but others may potentially produce other interactions. Extrapyramidal adverse effects have been reported in patients receiving both AChEI and antipsychotic agents, while those reactions did not occur when administered alone [92]. The same type of interaction could potentially occur between metoclopramide and AChEI because of its dopaminolytic properties [92]. Apart from the above mentioned effects, there is no data suggesting other drug–drug interactions between AChEIs and agents that are often used in anesthesiology and critical care.

### 8.2. Memantine

Memantine is an uncompetitive antagonist with moderate affinity for NMDA receptor channels. Persistent activation of NMDA receptors in the CNS has been hypothesized to contribute to the symptomatology of Alzheimer’s disease [93]. There is no evidence that memantine slows down neurodegeneration in patients with AD [93].

Conditions that raise urine pH may decrease urinary elimination of memantine resulting in its increased plasma levels. These include using medications that make the urine alkaline such as sodium bicarbonate. Memantine is rather well tolerated and the most common adverse reactions that occur in this therapy are: dizziness, headache, confusion, and constipation [94]. The overall profile of adverse reactions was not different from the profile for the overall dementia population and they usually have mild to moderate severity. The combined use of memantine with other NMDA antagonists like amantadine, ketamine, and dextromethorphan has not been systematically evaluated and such use is discouraged. Although there is no data indicating the time at which memantine should be discontinued, prior to administration of the above mentioned drugs, based on memantine elimination half-life, one should consider discontinuing memantine for 2–3 weeks prior to surgery, if ketamine is likely to be used.

## 9. Delirium Management

Delirium is associated with an increased risk of death, mainly because of a higher risk of difficult weaning leading to chronic respiratory insufficiency with prolonged mechanical ventilation, higher risk of infections, and a plethora of consequences of immobility—all have significant impact on length of hospital stay. In survivors, delirium increases the risk of impaired cognitive function, even months after hospitalization. It has also been reported that delirium can be avoided in almost 40% of cases [95] and, therefore, management of the disease should always be approached in accordance to the sentence “prevention is better than a cure.”

In critically ill adults, the first step to prevent delirium is adequate pain management. It has been reported that over 50% of ICU patients experience pain during their hospitalization [88]. Pain is a very clinically important symptom in general, and should never be ignored. It is all the more important in cases of possible delirium because pain also contributes heavily to the development of delirium itself. In order to assess occurrence and severity of pain, it is recommended for a clinician to use one of the validated pain scales. For patients who can self-report pain, i.e., in the postoperative period, the Numeric Rating Scale (NRS) [90] can be used, whereas for unconscious and unable-to-communicate individuals in the ICU, the Critical Care Pain Observational Tool (CPOT) and Behavioral Pain Scale (BPS) can be implemented [96]. The guidelines of the Society of Critical Care Medicine (SCCM) recommend that adequate pain treatment should be administered before inducing sedation. Doing so lowers the effective dose of a sedative drug and ensures that the patient receives satisfactory analgesia [97]. Pain drugs should be routinely administered in the presence of NRS >4 points or BPS >5 points, or CPOT >3 points and before pain-inducing procedures, i.e., chest tube removal, arterial line placement, and wound drain removal [88]. In order to maintain effective analgesia, patient-controlled analgesia (PCA) has been recommended to be implemented in all cases when severe pain is possible or likely to occur.

Even in times of opioid-free anesthesia, opioids should be administered as a first-line pain treatment in critically ill adults [88]. Nevertheless, the dose of these agents should always be titrated to the lowest effective value in order to limit the number of side effects associated with opioid use and to prevent withdrawal syndrome. In order to lower the dose of opioids, it is recommended that adjunctive drugs are administered [98]. Among such non-opioid agents, acetaminophen and metamizole can be used. Additionally, low-dose ketamine can be added to the multicomponent pain management. It is important to note that non-steroid anti-inflammatory drugs should not be routinely chosen as adjunctive agents for pain treatment, mainly because of the risk of kidney injury and gastrointestinal bleeding. Contrarily to many perioperative guidelines, intravenous lidocaine is not recommended for routine use in critically ill adults because of potential cardiac and neurological toxicity [97]. When it comes to treating neuropathic pain, drugs such as gabapentin, carbamazepine and pregabalin, together with opioids, are recommended [97,98]. Lastly, regional anesthesia can be an effective choice for surgical patients.

Another important method of preventing the onset of delirium is to avoid drugs that can cause or aggravate delirium, which are agents with proven anticholinergic activity. This activity is assessed by the Anticholinergic Cognitive Burden (ACB) scale—drugs with score 1 are possibly anticholinergic, while those with scores 2 and 3 are definitely anticholinergic [99]. Each definite anticholinergic increases the risk of cognitive impairment by 46% within 6 years from drug’s administration [100]. The score of ACB scale is added together and each score ≥3 is associated with an increased risk of cognitive disorders and increased mortality. For each additional point in the total ACB score, there is a decrease of 0.33 MMSE over 2 years from drug administration [101]. It is worth noting that among drugs with a possible cholinergic effect, medication such as warfarin, furosemide, and prednisone are distinguished and are often used in the elderly [102].

Delirium prevention also includes reducing anxiety, decreasing the stress of being mechanically ventilated, and preventing agitation in those critically ill patients who require sedation [97]. It is now known that excessive sedation leads to respiratory depression and increased weaning time, cognitive decline, muscle weakness, immunosuppression, prolonged ICU stays, hemodynamic instability, and most importantly, delirium [98]. It is, therefore, of paramount importance to decrease the level of sedation from a deep state of sedation (RASS −3 and below) to a light one (RASS −2 to 0). However, in some cases, deep sedation is still required, i.e., patients with ARDS requiring decreasing patient-ventilator dyssynchrony and increasing lung compliance, patients with brain injury with an increased intracranial pressure, patients with status epilepticus and patients receiving neuromuscular blocking agents. Apart from the above mentioned examples, the choice is always to implement light sedation to reduce sedation-related side effects. The light sedation should focus on ensuring that the patient feels comfortable, cooperative, and calm (the so-called 3C-rule) [103]. This kind of sedation enables patients to maintain eye contact, interaction, and not to spontaneously fall asleep when uninterrupted [98]. In order to maintain light sedation, either titrating sedative agents to low doses or daily sedative interruptions are recommended [104,105,106]. For the choice of sedative drugs, SCCM recommends using either propofol or dexmedetomidine. However, the PRODEX (Dexmedetomidine Versus Propofol for Continuous Sedation in the Intensive Care Unit) study shows that dexmedetomidine is associated with lower incidence of delirium compared to propofol after cessation of sedation [107]. Nonetheless, it is important to note that both of these agents are more associated with an improved outcome (e.g., decreased incidence of delirium, shorter time on ventilator, shorter extubation time) than benzodiazepines and, therefore, the use of benzodiazepines should be limited to a bare minimum. According to the eCASH concept, benzodiazepines can only be administered in cases of alcohol withdrawal syndrome, procedural sedation, severe brain pathologies, intractable agitation, and palliation [98].

When delirium is diagnosed with one of the validated screening tools, e.g., CAM-ICU, a clinician has to seek the potential causes of the disease. Handling the causes, such patient-ventilator dyssynchrony or anxiety, could effectively reduce the duration of agitation. It is important to note that prophylactic use of antipsychotics to prevent delirium is discouraged [98,108]. Additionally, antipsychotics (e.g., haloperidol) should not stand as a first-line treatment of delirium. Instead, SCCM recommends choosing dexmedetomidine as a therapy for mechanically ventilated adults, where agitation precludes extubation. Notably, according to the NICE (National Institute for Health and Care Excellence) guidelines, when a patient with delirium is agitated to a point where they pose a threat to the medical staff, and other techniques of de-escalation are ineffective, a short-term haloperidol usage is proposed (1 week of less) [96].

In order to prevent or reduce the duration of delirium, it should be remembered that non-pharmacological strategies can play a large role. It has been advised that promoting sleep hygiene and ensuring proper circadian rhythm can effectively improve outcomes in critically ill patients [88,97,98]. Reducing noise and light (by using eye shades and earplugs) during the night could help in achieving this goal [103,109]. Additionally, introduction of early mobilization and physiotherapy helps to maintain muscle strength, reduce disability, and prevent and treat delirium [110].

Most of the above recommendations also emphasize the role of humane care and family engagement [88,96,103]. The presence of loved ones and frequent communication with patients can help them cope with the stress associated with an ICU stay, reduce anxiety, and therefore maintain cognitive balance. Inclusion of family members helps to complete the treatment process, as it incorporates their wishes and concerns, which is beneficial for all of the treatment elements [88].

## 10. Summary

Delirium is a life-threatening condition, the causes of which are still not fully understood. It may develop in patients with pre-existing dementia. DSD can go completely unnoticed with routine examination. It may happen in the perioperative period and in the ICU setting, especially in the ageing population. Difficulties in diagnosing and lack of specific pharmacological and non-pharmacological treatment make DSD a seriously growing problem. Patient-oriented, multidirectional preventive measures should be applied to reduce the risk of DSD. For this reason, anesthesiologists and intensive care specialists should be aware of this interesting condition in their everyday clinical practice.

## Figures and Tables

**Table 1:** Vulnerability and precipitating factors for delirium [23,24,27,28,29].

Vulnerability Factors	Precipitating Factors
Demographics male gender age ≥ 65 years old educational background	Severe illness infection, sepsis inadequate pain control trauma hypo- or hyperthermia
Comorbidity dementia chronic kidney disease endstage liver disease terminal illness	CNS illness intracerebral hemorrhage cerebrovascular accident meningitis/encephalitis nonconvulsive status epilepticus cerebral edema tumor hydrocephalus
Functional status immobility visual impairment hearing impairment	Metabolic disorder thiamine deficiency renal failure liver failure electrolyte imbalance hypo- or hyperglycemia thyroid dysfunction glucocorticoid therapy hypophysis disfunction porphyria
Baseline medication usage polypharmacy baseline psychoactive medication and drug use * substance withdrawal	Cardiorespiratory acute coronary disease congestive cardiac failure hypoxemia hypercarbia shock hypertensive encephalopathy
Malnutrition & dehydration	Mobility restriction use of physical restraints use of bladder catheters intubation assisted ventilation use of vascular catheters intermittent pneumatic compression orthopedic cast
Depression	Other malignant hyperthermia serotonin syndrome malignant catatonia paraneoplastic syndrome >3 medications added anti-NMDA(*N*-methyl-d-aspartate) encephalitis any iatrogenic event drugs and medications taken
Circadian rhythm disruption	

* such as: benzodiazepines, opioids, medication with anticholinergic properties, alcohol, cocaine, phencyclidine, LSD, hallucinogenic substances. CNS, central nervous system.

**Table 2:** Comparison of delirium and dementia [58,59].

Feature	Delirium	Dementia
Onset	Sudden—hours/days	Insidious and slow
Cause	Other medical emergency	Baseline CNS disease—neurodegenerative or other
Course	Can be short if treated; usually reversible	Progressive—treatment slows the progression
Attention	Impaired initially	Usually preserved; may be impaired in advanced stages
Orientation	Impaired initially	Usually preserved; may be impaired in advanced stages
Memory	Impaired initially; may be unable to recall the incident	Initially lost of short-term memory; degree of memory loss increases as the disease progresses
Behaviour	Agitated/somnolent	Usually normal; may become agitated in advanced stages
Perceptual Disturbances	Visual hallucinations; misperceptions; illusions	Hallucinations and misperceptions may occur mostly in DLB

DLB, dementia with Lewy bodies.

**Table 3:** Comparison of hypo- and hyperactive delirium [58].

Feature	Hypoactive	Hyperactive
Arousal	Decreased arousal and alertness; somnolence; reduced awareness	Hypervigilant; easily startled; distractable
Mood	Depressed, irritable; mood swings; patient is disinhibited	Labile: from comative to euphoric
Psychomotor activity	Slow, quiet, withdrawn	Restless, agitated, combative, irritable
Past psychiatric history	May have experienced delirium before	Correlated with alcohol or drug withdrawal; may have experienced delirium before
Circadian rythm	Increased daytime sleepiness	Prominent disturbances; nightmares and night terrors

**Table 4:** Differential diagnosis of delirium superimposed on dementia (DSD) [78].

	Disease	Diagnostic Method
**CNS**	stroke, trauma brain injury, subsclerotic haemorrhage	CT/MRI
	Non-convulsive status epilepsy, partial epileptic seizure with cognitive decline	EEG
**Cardiovascular System**	Acute coronary syndrome	ECG, Troponin, CK-MB
	Atrial fibrillation	ECG
	Abdominal aortic aneurysm	Abdominal ultrasound, CT, MRI
**Respiratory System**	Pulmonary embolism	CT, angiography of pulmonary vessels, ventilation/perfusion scintigraphy
	Pulmonary oedema	Chest radiograph
**Metabolic Disturbances**	Hypermetabolic crisis, myxoedema	TSH, fT4
	Diabetes mellitus—keto alkalosis Hyperosmolar Hyperglycaemic Nonketotic Syndrome (HHNS)	Glycaemia measurement
	Acid-Base balance disturbances	Arterial blood gas
	Electrolyte balance disturbances; dehydration	Plasma electrolytes measurement
**Hematologic Disturbances**	Acute blood loss	Blood morphology evaluation
**Medication**	benzodiazepines, antipsychotics, opioids, sedation drugs, anticholinergic drugs	Drug level in blood/urine
**Other**	Sleep deprivation, psychosis, depression	Psychiatric evaluation

CT—Computed Tomography, MRI—Magnetic Resonance Imagining, ECG—Electrocardiography, EEG—Electroencephalography.
